# Comparison of GRACE, HEART, and TIMI Risk Scores in Predicting In-Hospital Morbidity and Mortality in Acute Coronary Syndrome

**DOI:** 10.7759/cureus.115009

**Published:** 2026-08-23

**Authors:** Kaleemullah Shaikh, Abeer Sarfaraz, Nida Batool, Fazila Sikandar, Shafaq Saher, Fahad Kaleem

**Affiliations:** 1 Department of Cardiology, Liaquat National Hospital and Medical College, Karachi, PAK; 2 Department of Dentistry, Ziauddin University, Karachi, PAK

**Keywords:** acute coronary syndrome, grace score, heart score, in-hospital mortality, major adverse cardiovascular events, timi score

## Abstract

Background

Uncertainty persists regarding the comparative predictive value of various risk scores for in-hospital outcomes in patients with acute coronary syndrome (ACS).

Methodology

A total of 259 patients with ACS were included in this study. Clinical and electrocardiographic data were collected, and in-hospital mortality and morbid events were documented. Using multivariate logistic regression analysis, predictors were identified. The discriminatory ability of the Global Registry of Acute Coronary Events (GRACE), Thrombolysis in Myocardial Infarction (TIMI), and HEART (History, Electrocardiogram, Age, Risk factors, and Troponin) scores was determined using receiver operating characteristic curve analysis and compared using the DeLong test. Calibration was checked using the Hosmer-Lemeshow goodness-of-fit test.

Results

Hospital deaths were noted in 20 (7.7%) cases, and hospital morbidities in 61 (23.6%) cases. Significant predictors of mortality included older age, ST-segment changes, T-wave inversion, and decreased percutaneous coronary intervention. Predictors of morbidity included older age, lower body mass index, chronic kidney disease, and elevated creatinine level. Regarding multivariate analysis, age (odds ratio (OR) = 1.086; 95% confidence interval (CI) 1.005 to 1.173; p = 0.036) and TIMI score (OR = 1.542; 95% CI = 1.100 to 2.163; p = 0.012) were found to be independent predictors of mortality, whereas only the TIMI score was an independent predictor of morbidity (OR = 1.933; 95% CI = 1.512 to 2.472; p < 0.001). The TIMI score showed better discrimination than both the GRACE and HEART scores, which had area under the curve values of 0.728 for mortality and 0.737 for morbidity.

Conclusions

The TIMI risk score demonstrated superior predictive performance compared with GRACE and HEART scores for in-hospital complications in patients with ACS.

## Introduction

Cardiovascular diseases are the leading cause of death, contributing to about 17.9 million deaths per year and constituting one-third of all global deaths [1]. Current evidence from the Global Burden of Disease and the World Health Organization still shows a heavy burden of ischemic heart disease, especially in low- and middle-income countries, which have significantly elevated rates of premature cardiovascular deaths [2]. Acute coronary syndrome (ACS) can be considered a clinical presentation of ischemic heart disease and is characterized by significant morbidity and mortality. ACS includes unstable angina, non-ST-elevation myocardial infarction (NSTEMI), and ST-segment elevation myocardial infarction (STEMI). It is diagnosed based on clinical presentation, ECG findings, and cardiac biomarkers, such as high-sensitivity troponins [3]. It is vital to identify early risk stratification in ACS, as it helps in decision-making, optimal resource utilization, and better patient outcomes.

Current international guidelines still emphasize the use of valid risk stratification tools for decision-making and predicting the prognosis of ACS patients [4]. Many validated risk stratification tools have been established and integrated in clinical practice. Of these, the Global Registry of Acute Coronary Events (GRACE) score, Thrombolysis in Myocardial Infarction (TIMI) score, and HEART (History, Electrocardiogram, Age, Risk factors, and Troponin) score are some of the frequently used stratification tools [5]. The GRACE risk score, based on the multinational registry data, shows excellent predictive validity for both short- and long-term mortality [6]. The TIMI score has remained popular owing to its simplicity and bedside availability [7]. The HEART score, which was designed mainly for use in the emergency room, has been proven to be effective in identifying low-risk patients who could safely be discharged early [8]. While these risk scores are commonly used, conflicting evidence exists on their predictive power. Recent studies and meta-analyses have shown discriminatory powers of the GRACE, TIMI, and HEART scores to be variable in terms of patient cohort, clinical setting, and clinical outcome [9,10]. Superior predictive performance of the GRACE score for mortality in different settings has been demonstrated by several studies, while others have underscored the usefulness of the HEART score in ruling out major adverse cardiovascular events with high negative predictive value. Additionally, the performance of the TIMI score has also been inconsistent in recent studies [11].

Of particular note, most of these studies are conducted in a Western setting with poor South Asian participation. This is clinically important as patients from South Asia exhibit unique risk characteristics such as young age, presence of metabolic syndrome, late presentation, and different treatment strategies [12]. Moreover, the rising incidence of ACS in Pakistan and other neighboring countries has also been recently shown in regional registries. These factors can affect the performance and discriminatory powers of ACS risk scores in clinical practice. Evidence comparing the prognostic performance of the GRACE, TIMI, and HEART risk scores in Pakistani patients with ACS is scarce. Hence, this study aimed to compare the predictive accuracy of these three risk stratification tools for in-hospital mortality and morbidity in this population. We hypothesized that one of the GRACE, HEART, or TIMI scores would provide superior discrimination for predicting adverse in-hospital outcomes.

## Materials and methods

The prospective cohort observational study was performed in the Department of Cardiology of a tertiary care teaching hospital from July 2025 to May 2026. Non-probability consecutive sampling was applied for the recruitment of patients aged ≥18 years presenting with ACS. The research was approved by the institutional ethics committee (approval number: RC.-LNH-cardiology-7/2025/76), adhering to the principles of the Declaration of Helsinki.

Consecutive adult patients (≥18 years) admitted with a diagnosis of ACS, including STEMI, NSTEMI, and unstable angina, were enrolled. Patients with complete clinical, ECG, laboratory, and outcome data necessary for the calculation of GRACE [6], TIMI [7], and HEART [8] risk scores were included. Patients younger than 18 years, those with non-ACS chest pain, incomplete medical records, missing variables required for risk score calculation, transfer from other institutions after initial ACS management, or discharge before outcome assessment were excluded. Randomization being not relevant in an observational study design, the assessors of the outcomes were blinded to risk scores, and vice versa.

Baseline demography, cardiovascular risk factors, presenting features, laboratory data, ECG findings, and treatment modalities were recorded using a pre-defined standardized proforma with double data entry methods. Predictive variables needed for calculation of GRACE, HEART, and TIMI scores were identified at the index presentation visit, and scoring was performed using the validated algorithms. The patients were followed up to discharge during the index hospitalization.

The two primary outcomes measured included mortality and morbidity during the hospital stay. In this study, mortality was defined as death that occurred during the hospital stay. On the other hand, morbidity was considered the occurrence of adverse clinical events such as recurrent myocardial infarction, stroke, arrhythmia, heart failure, stent thrombosis, and major bleeding while in the hospital. Outcomes were determined through review of medical records, daily clinical assessments, laboratory investigations, imaging studies, and discharge summaries by the treating physicians.

Sufficient sample size was determined based on the event-per-variable rule to guarantee model stability. There were very few missing values in this study, which were non-differential in nature; thus, complete-case analysis was adopted for analysis. Statistical analyses were performed using SPSS Statistics version 27 (IBM Corp., Armonk, NY, USA). Continuous variables were evaluated for normality using the Shapiro-Wilk test. Normally distributed continuous variables were presented as mean ± SD, while continuous variables that did not follow a normal distribution were presented as median (interquartile range (IQR)). Continuous variables were analyzed using an independent-samples t-test/Mann Whitney U test, depending on the distribution, while categorical variables were analyzed using Pearson chi-square/Fisher’s exact test. The predictive validity of GRACE, HEART, and TIMI risk scores was evaluated using receiver operating characteristic (ROC) curve analysis, and the discrimination power of each score was expressed using the area under the curve (AUC) along with its 95% confidence interval (CI). Comparison of the AUC of each score was done using the DeLong test. The presence of independent predictors of hospital outcome was determined using multivariate logistic regression, and the odds ratio (OR) with its 95% CI was computed. The presence of multicollinearity among variables was checked using the variance inflation factor, while the linearity assumption was tested using the Box-Tidwell test. Calibration of the model was checked using the Hosmer-Lemeshow test. Other diagnostic parameters such as sensitivity, specificity, positive predictive value, negative predictive value, and accuracy were also obtained. A two-tailed p-value <0.05 was considered statistically significant. Variables demonstrating statistical significance in the univariable analysis (p < 0.05), together with clinically important variables identified from previous literature, were entered into the multivariable logistic regression models.

## Results

**Figure 1:**
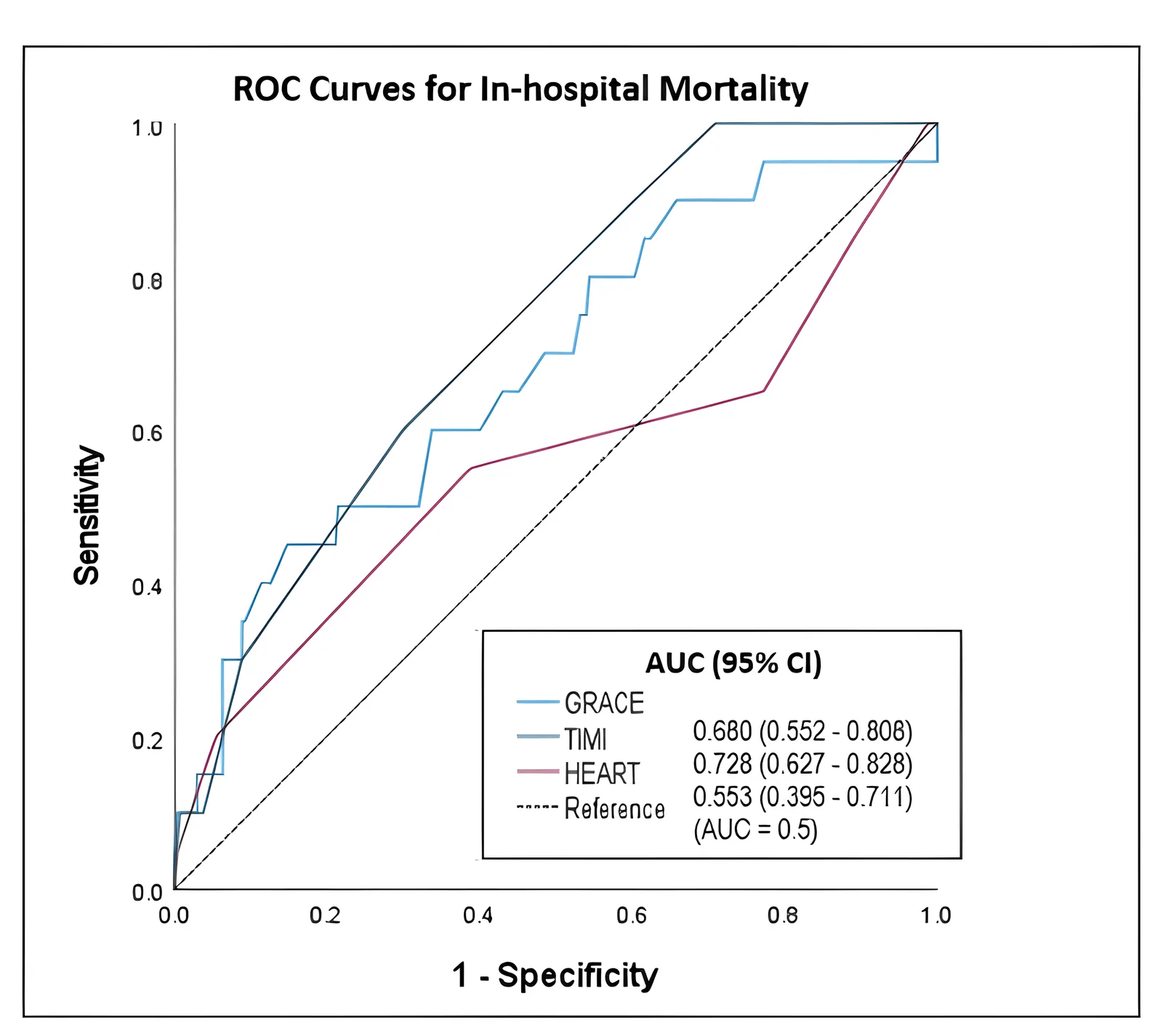
Receiver operating characteristic curves for the prediction of in-hospital mortality using GRACE, TIMI, and HEART scores. AUC = area under the curve; CI = confidence interval; GRACE = Global Registry of Acute Coronary Events; HEART = History, Electrocardiogram (ECG), Age, Risk factors, and Troponin; TIMI = Thrombolysis in Myocardial Infarction

**Figure 2:**
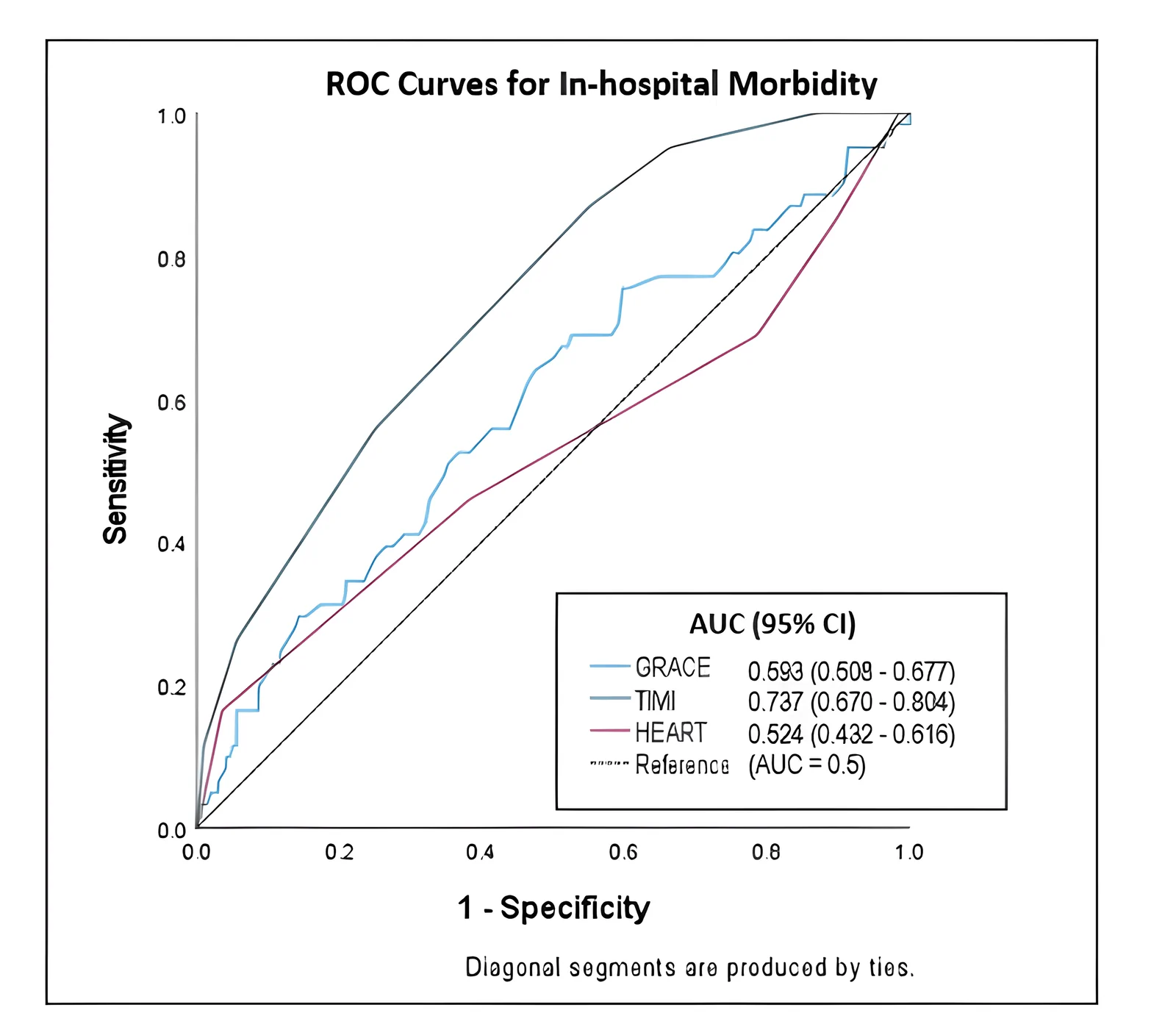
Receiver operating characteristic curves depicting the discriminative ability of GRACE, TIMI, and HEART scores in predicting in-hospital outcomes in patients with ACS. ACS = acute coronary syndrome; AUC = area under the curve; CI = confidence interval; GRACE = Global Registry of Acute Coronary Events; HEART = History, Electrocardiogram (ECG), Age, Risk factors, and Troponin; TIMI = Thrombolysis in Myocardial Infarction

In the current study, a total of 259 patients were considered, of whom 20 (7.7%) died within the hospital premises, while 61 (23.6%) experienced morbidity. The age of the deceased was greater than that of surviving patients, who showed less frequency of ST-segment elevation, more T-wave inversion, and percutaneous coronary intervention was less common among them compared to survivors (Table 1). The group facing morbidity had greater age, lower BMI, higher prevalence of chronic kidney disease, and increased serum creatinine level (Table 1). Recurrent myocardial infarction and heart failure emerged to be independent predictors for mortality, while recurrent myocardial infarction, stroke, arrhythmia, heart failure, and stent thrombosis were independent predictors for morbidity (Table 2). According to ROC analysis, there is better discrimination for the TIMI score compared to the GRACE and HEART scores regarding mortality and morbidity prediction (Table 3, Figure 1, Figure 2).

**Table 1:** Baseline demographic and clinical characteristics according to in-hospital mortality and morbidity. Values are presented as n (%) or median (IQR). Categorical and continuous variables were compared using the chi-square/Fisher’s exact test and Mann–Whitney U test, respectively. Statistical significance was set at p < 0.05. BMI = body mass index; BP = blood pressure; CKD = chronic kidney disease

Variable	Mortality		U(z)/χ² (df)	P-value	Morbidity		U(z)/χ² (df)	P-value
	Yes (n = 20), n (%)	No (n = 239), n (%)			Yes (n = 61), n (%)	No (n = 198), n (%)		
Age, years	71.5 (6.0)	65.0 (13.0)	1,177 (-3.774)	<0.001	68.0 (10.0)	64.0 (14.0)	4,678.5 (-2.663)	0.008
BMI, kg/m²	25.6 (5.1)	27.6 (4.3)	1,830.5 (-1.739)	0.082	26.6 (5.2)	27.7 (4.2)	4,923.5 (-2.181)	0.029
Male sex	3 (15.0%)	85 (35.6%)	3.479 (1)	0.062	41 (67.2%)	130 (65.7%)	0.050 (1)	0.822
Diabetes mellitus	11 (55.0%)	167 (69.9%)	1.900 (1)	0.168	38 (62.3%)	140 (70.7%)	1.535 (1)	0.215
Hypertension	13 (65.0%)	191 (79.9%)	2.455 (1)	0.151	46 (75.4%)	158 (79.8%)	0.537 (1)	0.464
CKD	6 (30.0%)	36 (15.1%)	3.031 (1)	0.108	16 (26.2%)	26 (13.1%)	5.889 (1)	0.015
Systolic BP, mmHg	137 (13)	130 (22)	2,119.50 (-0.844)	0.399	138 (13)	130 (24)	4,609 (-2.807)	0.005
Creatinine, mg/dL	1.21 (1.52)	1.10 (0.63)	2,239.50 (-4.68)	0.64	1.33 (1.46)	1.08 (0.52)	4,817 (-2.389)	0.017
ST-elevation	5 (25.0%)	151 (63.2%)	11.231 (1)	0.001	20 (32.8%)	136 (68.7%)	25.091 (1)	<0.001
ST-depression	14 (70.0%)	183 (76.6%)	0.437 (1)	0.585	38 (62.3%)	159 (80.3%)	8.305 (1)	0.004
T-wave inversion	11 (55.0%)	73 (30.5%)	5.037 (1)	0.025	32 (52.5%)	52 (26.3%)	14.604 (1)	<0.001
Killip Class IV	4 (20%)	5 (2.1%)	Fisher’s exact	0.003*	5 (8.2%)	4 (2%)	Fisher’s exact	0.036*
Length of stay (days)	5.5 (1.0)	4.0 (2.0)	4,126.5 (5.666)	<0.001*	5.0 (1.0)	4.0 (1.0)	10,196 (8.532)	<0.001*

**Table 2:** In-hospital clinical outcomes according to mortality and morbidity. Values are presented as n (%). Comparisons were performed using Fisher’s exact test, as appropriate. Statistical significance was set at p < 0.05.

Outcome	Mortality		Test statistics	P-value	Morbidity		Test statistics	P-value
	Yes (n = 20), n (%)	No (n = 239), n (%)			Yes (n = 61), n (%)	No (n = 198), n (%)		
Myocardial infarction	7 (35.0%)	14 (5.9%)	Fisher’s exact	<0.001	21 (34.4%)	0 (0%)	Fisher’s exact	<0.001
Stroke	2 (10.0%)	12 (5.0%)	Fisher’s exact	0.295	14 (23.0%)	0 (0%)	Fisher’s exact	<0.001
Arrhythmia	1 (5.0%)	5 (2.1%)	Fisher’s exact	0.386	6 (9.8%)	0 (0%)	Fisher’s exact	<0.001
Heart failure	3 (15.0%)	8 (3.3%)	Fisher’s exact	0.044	11 (18.0%)	0 (0%)	Fisher’s exact	<0.001
Stent thrombosis	0 (0%)	4 (1.7%)	Fisher’s exact	>0.999	4 (6.6%)	0 (0%)	Fisher’s exact	<0.001

**Table 3:** Performance, calibration, and comparative accuracy of GRACE, TIMI, and HEART scores for in-hospital outcomes. Values are presented as AUC with 95% CIs, optimal cutoff values, sensitivity, specificity, PPV, NPV, and overall accuracy. Calibration was assessed using the Hosmer–Lemeshow goodness-of-fit test; p > 0.05 indicates adequate model calibration. AUC = area under the curve; CI = confidence interval; GRACE = Global Registry of Acute Coronary Events; HEART = History, Electrocardiogram (ECG), Age, Risk factors, and Troponin; PPV = positive predictive value; NPV = negative predictive value; TIMI = Thrombolysis in Myocardial Infarction

Outcome	Score	AUC	Cutoff	Sensitivity	Specificity	PPV	NPV	Accuracy	Hosmer–Lemeshow p-value
		(95% CI)							
Mortality	GRACE	0.68	>140	50.00%	76.20%	14.90%	94.80%	74.10%	0.421
		(0.556–0.804)							
	TIMI	0.728	≥5	30.00%	91.20%	22.20%	93.90%	86.40%	0.538
		(0.612–0.844)							
	HEART	0.553	≥7	65.00%	22.80%	6.60%	88.50%	26.10%	0.611
		(0.418–0.688)							
Morbidity	GRACE	0.593	>140	34.40%	76.80%	31.30%	79.20%	74.50%	0.332
		(0.513–0.672)							
	TIMI	0.737	≥5	26.20%	94.40%	59.30%	80.50%	78.30%	0.447
		(0.668–0.806)							
	HEART	0.524	≥7	68.90%	21.40%	21.40%	68.90%	32.70%	0.518
		(0.445–0.603)							

On multivariate logistic regression analysis, age (OR = 1.086; 95% CI = 1.005-1.173, p = 0.036) and TIMI score (OR = 1.542; 95% CI = 1.100-2.163, p = 0.012) were identified as independent predictors of mortality, while TIMI demonstrated the highest discrimination, although overall discrimination remained moderate (OR = 1.933, 95% CI = 1.512-2.472, p < 0.001) (Table 4). According to DeLong analysis, there was significantly better discrimination for morbidity but not for mortality prediction using the TIMI score compared with the GRACE and HEART scores (Table 4). In addition, calibration analysis indicated satisfactory goodness-of-fit for the three risk scoring tools, with non-significant results from the Hosmer-Lemeshow test for both mortality and morbidity outcomes (all p > 0.05) (Table 3).

**Table 4:** Multivariable logistic regression analysis for in-hospital outcomes. Values are presented as ORs with 95% CIs. An OR >1 indicates increased odds of the outcome per unit increase in the predictor variable. Statistical significance was defined as p < 0.05. BMI = body mass index; CI = confidence interval; TIMI = Thrombolysis in Myocardial Infarction

Variable	Mortality	Morbidity	Interpretation
	OR (95% CI)	OR (95% CI)	
Age	1.086 (1.005–1.173)	1.020 (0.977–1.064)	Significant predictor for mortality only
BMI	0.882 (0.761–1.022)	0.910 (0.827–1.002)	Not statistically significant
TIMI score	1.542 (1.100–2.163)	1.933 (1.512–2.472)	Strong independent predictor of both outcomes

## Discussion

The main objective of this prospective observational study on ACS patients at a single medical center was to assess the discriminatory accuracy of the GRACE, TIMI, and HEART scores in terms of their prediction of in-hospital mortality and morbidity. The primary finding of the present study is the higher discriminatory ability of the TIMI score in comparison to the GRACE and HEART scores, especially regarding morbidity. The GRACE score had moderate discriminatory ability, while the HEART score had poor discriminatory ability.

The demographics of the cohort were consistent with previous registries of ACS in South Asians, in particular with younger patient age and preponderance of males when compared to other geographic regions [13]. Older age was independently associated with mortality in the cohort, highlighting its persistent value as a risk factor for ACS-related poor outcomes even in light of recent advancements in management strategies [14]. Males had a higher incidence of complications when compared with females in this cohort, despite evidence of poorer outcomes in women with ACS. Such inconsistencies might be attributed to differences in access to healthcare, delay in treatment, or burden of comorbidities [15].

When comparing the predictive capabilities of the various risk stratification scores used in the cohort, the TIMI score performed the best overall, having adequate discriminatory power regarding mortality and morbidity while being independently associated with the presence of negative events [16]. Similar results regarding TIMI’s predictive power have been observed in previous studies evaluating South Asians with ACS, where it was noted that simpler bedside scoring systems exhibited some degree of prognostic usefulness in resource-limited areas. The better performance of TIMI may be due to its use of easily identifiable clinical criteria directly correlated with acute ischemia risk.

Although the GRACE risk score had moderate discriminatory power for mortality, it was not an independent predictor when other covariates were adjusted. This can be attributed to the overlap of variables present in both TIMI and GRACE risk scores, which reduces the added value of the latter risk score in the multivariable analysis. On the other hand, a number of multicenter registries have found that the GRACE score discriminates mortality better compared to the TIMI score, especially among higher-risk groups [17]. One possible reason for this is that the GRACE score has been developed in a more diverse multinational population setting with more significant event rates than the current sample used in this study.

Despite being a better predictor, the TIMI score possesses several limitations. First, compared to the GRACE risk score, it does not contain several high-risk variables that have been identified to be significant predictors of prognosis, including heart rate, hemodynamic instability, cardiac arrest, and serum creatinine [18,19]. Second, use of only TIMI risk score-based classification may result in underestimation of risk in some patients. However, while GRACE offers much more accurate discrimination, it is also complicated and uses a calculator for its computation.

Performance of the HEART score as a predictor of adverse events was found to be inadequate in the current study. HEART was mainly validated in undifferentiated emergency department chest pain populations, whereas ours was hospitalized confirmed ACS [20]; its inability to predict adverse events in hospitalized ACS patients could be due to its intended purpose as a short-term stratifier of risk.

Interestingly, the difference between various risk scores regarding predictive performance was more evident with regard to morbidity than mortality outcomes. It appears that discrimination of mortality is less affected by the specific algorithm employed in risk stratification, compared with morbidity, given that the TIMI score outperformed both the GRACE and HEART scores when predicting morbidity outcomes. Calibration was adequate for all three scores with insignificant values of the Hosmer-Lemeshow test, albeit a potential concern with regard to this particular test’s known vulnerability to sample size. Overall, these results point toward the need for existing ACS risk scores to be regarded as complements to the full clinical picture rather than a substitute. Proper risk assessment is probably only possible with the combination of well-known risk scores, physician discretion, procedural specifics, imaging, and biomarkers. Machine learning risk assessment approaches could enhance prognosis in future ACS patients.

This study has several limitations. It was a single-center study with a relatively small sample size, limiting statistical power due to the low number of deaths and reducing the stability of multivariable regression estimates. The absence of long-term follow-up also restricted outcome assessment to the in-hospital period. Therefore, external validation in larger, multicenter cohorts is warranted. The superior performance of the TIMI score may reflect its reliance on binary variables, including positive cardiac biomarkers and traditional cardiovascular risk factors, which are common in our cohort. In contrast, the HEART score includes subjective assessment of chest pain history and ECG interpretation, potentially introducing variability. The comparatively lower performance of the GRACE score may be explained by its original design to predict mortality, particularly over longer-term follow-up, whereas our study assessed in-hospital mortality and a broader composite morbidity endpoint.

## Conclusions

In patients with ACS, the TIMI score had better but still moderate discriminatory ability compared to the GRACE and HEART scores, especially regarding morbidity. Risk scoring models should serve as adjunctive aids to clinical evaluation. Future studies involving larger multicenter cohorts are needed to develop and validate contemporary population-specific risk prediction models.
